# Nonsecretory intestinocystoplasty: postoperative outcomes of 25 years

**DOI:** 10.1590/S1677-5538.IBJU.2018.0595

**Published:** 2019-09-02

**Authors:** Rose A. F. Dantas, Fernanda C. F. S. Calisto, Fabio O. Vilar, Luiz A. P. Araujo, Salvador V. C. Lima

**Affiliations:** 1 Programa de Pós-Graduação em Cirurgia, Centro de Ciências da Saúde, Universidade Federal de Pernambuco-UFPE, Pernanbuco, PE, Brasil;; 2 Departamento de Urologia, Hospital das Clínicas da Universidade Federal de Pernambuco-UFPE, Pernanbuco, PE, Brasil;; 3 Departamento de Cirurgia Pediátrica, Universidade Federal de Pernambuco-UFPE, Pernanbuco, PE, Brasil;; 4 Departamento de Cirurgia, Centro de Ciências da Saúde da Universidade Federal de Pernambuco-UFPE, Pernanbuco, PE, Brasil

**Keywords:** Hypopituitarism, Urinary Bladder, Disease

## Abstract

**Objective:**

The objective of bladder augmentation (BA) is to create a low-pressure reservoir with adequate capacity. Despite its benefits, the use of intestinal patches in bladder enlargement provides a high risk of developing complications and BA with demucosalised bowel represents a potential alternative. Therefore, this study evaluated urological parameters and long-term clinical follow-up of patients submitted to non-secretory BA in a single center with 25 years of experience.

**Materials and Methods:**

Patients treated with BA underwent urological evaluation, which included history, physical examination and urodynamic study. The main urodynamic parameters (bladder capacity and bladder compliance) were assessed in the pre and postoperative moments, and compared by the Wilcoxon Signed Rank test. The main long-term complications were described.

**Results:**

269 patients (mean age 14±13 years, 47% male) underwent BA with the use of demucolised intestinal segments. Among the patients in the sample, 187 (69.52%) had neurogenic bladder, 68 (25.28%) had bladder exstrophy, nine had tuberculosis (3.34%), four had a posterior urethral valve (1.49%) and one with hypospadia (0.37%). After the surgical procedure, a significant increment in both urodynamic parameters was found, with a 222% increase in bladder capacity and 604% in bladder compliance (p <0.001 in both analyzes). Mean follow-up time ranged from 2 to 358 months, with a median of 72 months (IQR 74-247). Among all patients, 5 presented spontaneous perforation.

**Conclusion:**

The study showed statistically significant increase in both compliance and bladder capacity after non-secretory BA, with a low rate of severe complications.

## INTRODUCTION

The use of intestinal segments in bladder augmentation has promoted important advances on finding new ways to deal with patients with noncompliant bladders. Nevertheless, there is a concern regarding specific characteristics of the intestinal epithelium that can result in complications in the medium and long-term follow-up, affecting quality of life and prognosis of the patients. Among the main drawbacks are calculus formation, mucus production, metabolic acidosis, urinary tract infections, intestinal obstruction and increased long-term risk of cancer (1, 2). On the other hand, non-secretory bladder augmentation may represent a potential alternative. This study is an update of our experience with demucolised bowel segments for bladder augmentation (3-5).

## MATERIALS AND METHODS

During a period of 25 years (January 1991-June 2016), all patients who underwent demucolised bladder enlargement in a single center were included in the study. This study was conducted after a detailed animal experiment (3, 4). Patients with age greater than 55 years and those who had malignant disease with a life expectancy of less than 10 years were excluded. The procedures for diagnosis and surgical technique have been described previously (3-6). All patients were submitted to urologic evaluation, which included medical history, physical exam and assessment of urodynamic parameters. Data on bladder capacity and compliance were used to evaluate results. The same equipment measured cystometric maximum bladder capacity and compliance. Expected bladder capacity according to the formula described by Houle et al. (7) was also used as a comparison. All procedures in the study were performed by or under the supervision of the first three authors, who have carried out the study from the experimental phase.

Among all surgical interventions, 222 (82.5%) were performed with sigmoid intestinal patches (Group-A) and 47 (17.5%) were performed using ileum intestinal patches (Group-B). Surgical intervention was performed as follows: In group A, the entire width of the bladder wall was opened, including the mucosa. In order to prevent retraction of the graft, a silicone bladder modeler was inserted inside the enlarged bladder. This model was filled with saline solution at volumes that ranged from 40 to 250mL and maintained for two weeks. During this same period, the ureters were catheterized. In group B, the process of de-epithelialization was facilitated by insertion of a Foley with a 30mL balloon inflated until 7-8mL. The amount of fluid within the balloon varied according to the characteristics of each patient’s pelvis.

Mean and standard deviation were used when there was normal distribution, and median and interquartile range (IQR) were used when distribution was non-normal. Normality of data was evaluated through histograms and the Shapiro-Wilk test. The Wilcoxon Sign-Rank test was used to compare if there was a statistically significant difference between preoperative and postoperative capacity and compliance. All statistical calculations were performed with SPSS software version 18.1®. In all situations, the maximum acceptable probability of error for rejection of the null hypothesis was 5% (p <0.05 and confidence interval of 95%). This study was approved by the Research Ethics Committee of the Center for Health Sciences, Federal University of Pernambuco (APPROVAL NUMBER: 2.430.399).

## RESULTS

**Figure 1:**
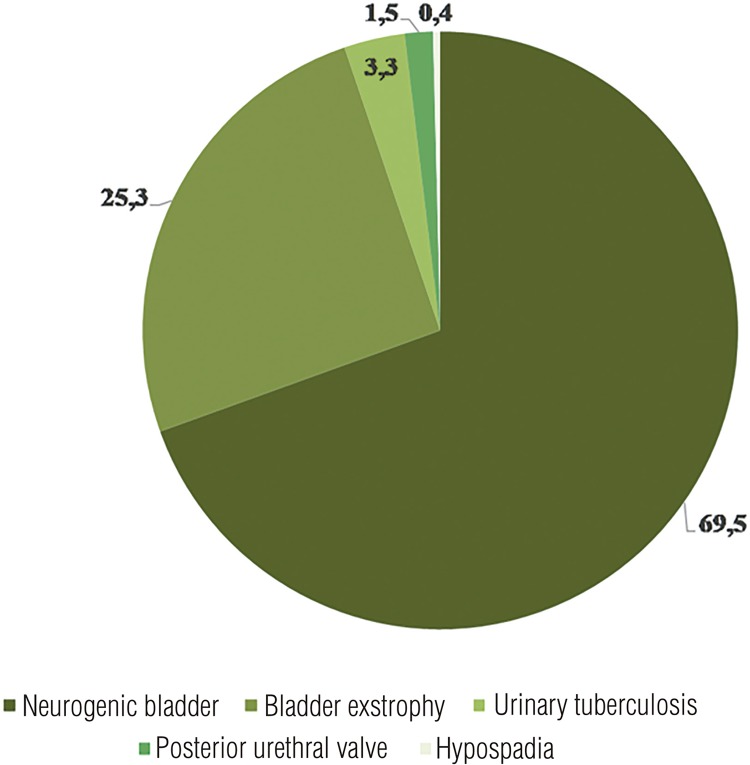
Diagnosis at preoperative assessment.

A total of 269 patients who had undergone bladder augmentation using de-epithelialized intestinal segments were prospectively studied between January 1991 and June 2016. Patient age varied from 3 months to 55 years, with mean age of 14±13 years, 47% male. Of these patients, 187 (69.5%) were diagnosed with neurogenic bladder, 68 (25.3%) had bladder exstrophy, 9 (3.3%) patients had been treated for urinary tuberculosis, 4 (1.5%) had sequelae of posterior urethral valves and 1 (0.4%) presented with female hypospadia (Figure-1). Ileum was chosen in cases of bladder exstrophy or when there was any problem with the colon (previous surgeries). Follow-up time varied from 2 to 358 months, with median follow-up of 72 months (IQR 74-247).

**Figure 2:**
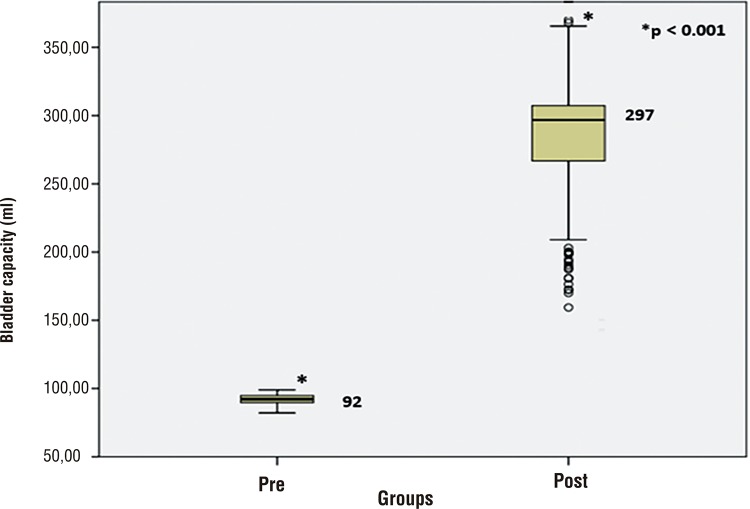
Comparison of preoperative and postoperative bladder capacity.

**Figure 3:**
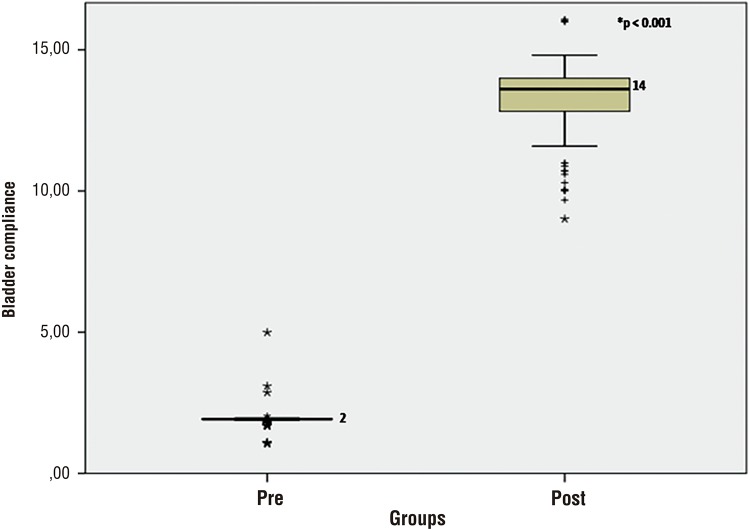
Comparison of preoperative and postoperative bladder compliance.

Median preoperative and postoperative bladder compliance was 1.93mL/cm/H20 (IQR 1.91-1.95) and 13.6mL/cm/H20 (IQR 12.8-13.9), respectively (p <0.001) (Figure-2). This resulted in an increment of 604%. Median preoperative and postoperative capacity was 92.1mL (IQR 90-95) and 296.8mL (IQR 265-308), respectively (p <0.01), resulting in an increment of 222% (Figure-3). The mean expected capacity by Houle’s formula was 269mL (6), lower than the postoperative capacity levels that were obtained in the sample.

A total of 27 patients presented some kind of complication (10%). Three patients (1.1%) developed peritonitis with complete dehiscence of the wound, 4 patients (1.4%) presented dehiscence of the colonic anastomosis and 5 patients (1.8%) presented spontaneous perforation. Throughout this period, 13 cases (4.8%) of bladder lithiasis were identified in patients with bladder exstrophy. In this group, 6 patients had a periurethral constrictor implanted simultaneously, and 5 of these were removed due to urethral erosion. Four patients in the exstrophy-epispadia group underwent post-void catheterization due to significant post-void residue. Spontaneous perforations were observed in patients diagnosed with bladder exstrophy and neurogenic bladder. The five cases of spontaneous perforation occurred at the region where the anastomosis was made between bladder and demucolised tissue. Out of all spontaneous perforations, 2 happened with ileum patches and 3 with sigmoid patches. The first and second cases had diagnoses of bladder exstrophy and perforation occurred after 5 and 7 years; the other 3 cases were observed in with neurogenic bladder; the perforation occurred after 2 years, 3 years and 2 years and 7 months, respectively. All three patients underwent intermittent catheterization through the urethra. During the 25 years follow-up of this group of patients, 11 deaths were verified; however, none of them were related to the bladder surgical procedure. Of the total sample, 35 cases (13%) were considered failures.

## DISCUSSION

The complications that are intrinsic to the traditional techniques of intestinal cystoplasty have made surgeons pursue alternative modalities of bladder augmentation over time. Non-secretory bladder enlargement has been presented as one of these alternatives, as it does not present the characteristic disadvantages of the secretory and absorptive function of the intestinal mucosa. To our knowledge there is no report in the literature of a prospective study of bladder augmentation for this period of time at the same institution and by the same team starting at experimental phase. Studies were published in periods of time around not below 5 years.

In the present study, we found a statistically significant increase in bladder compliance and capacity after non-secretory bladder augmentation. This result is in agreement with the current literature. In a recent study published by Odeh et al. (8), patients were compared regarding bladder augmentation techniques (traditional ileocystoplasty vs. ileocystoplasty with demucolised bowel) and followed for 14 years. Authors reported that there was an increase in bladder capacity in both groups (without significant intergroup difference), therefore supporting our results. Several other studies have demonstrated an increase in bladder capacity following non-secretory intestinal cystoplasty. Jung et al. (9) analyzed 34 patients who underwent demucolised intestinal cystoplasty with urothelial alignment. The authors described a 2.96-fold increase in bladder capacity, and a total of 13 patients (39.4% of the sample) were able to suspend the use of anticholinergic drugs after 47.3 months of surgery. In a study by Jednak et al. (10), the increase in bladder capacity was of 2.94 times.

Regarding the complications, we found an incidence of 1.8% of spontaneous perforations (5 cases), which is lower than the current literature statistics when compared with a similar method or with traditional ileocystoplasty. In a study by Shekarriz et al. (11), 133 patients underwent several types of bladder augmentation and were studied for a similar period. The authors found six cases of intestinal obstruction, 17 spontaneous perforations (13%), and 15 patients requiring surgical revision for other reasons (12). In a case-control study conducted by Odeh et al. (8), the authors reported an incidence of 10% of spontaneous perforations in the sample.

The incidence of bladder lithiasis was of 13 cases (4.8%), which represents a lower rate as compared to previous reports. Additionally, all cases happened in patients who had bladder exstrophy. In a retrospective study with 91 children that had undergone bladder reconstruction with various segments of the digestive tract over a period of 10 years, Hensle et al. (12) found an incidence of 44% of bladder lithiasis. Among patients with bladder exstrophy, 5 (25%) presented such situation. In a retrospective series performed by Blaivas et al. (13) with 71 patients submitted to enterocystoplasty, authors found 6% of recurrent bladder lithiasis. The bladder lithiasis cases accounted for 19% (13 of 68) of the sample of patients diagnosed with bladder exstrophy. It is worth mentioning that 6 patients who presented with bladder lithiasis in our cohort had a peri-urethral constrictor implanted simultaneously, 5 of which were removed due to urethral erosion. Since no more artificial devices are currently used in patients with a diagnosis of exstrophy, we expect this incidence to reduce, since it is known that procedures on the bladder neck increase the incidence of lithiasis. On the other hand, long-term follow-up has shown a high incidence of device erosion both at subcutaneous port and cuff site. A new intraurethral removable device has shown considerable improvements in continence, quality of life and occurrence of symptomatic urinary tract infections (UTI). This device has been applied to females but possibilities to its use in males are open (14).

Despite the visible improvements, we must recognize that a failure rate of 13% is still not ideal. New developing technologies may help improve these results in the future.

## CONCLUSION

During this 25 year follow-up study, non-secretory bladder augmentation promoted a significant increase in bladder compliance and capacity. As compared to traditional techniques of bladder augmentation, a lower number of complications was observed.

## References

[1] 1. Castellan M, Gosalbez R, Bar-Yosef Y, Labbie A. Complications after use of gastric segments for lower urinary tract reconstruction. J Urol. 2012;187:1823-7. 10.1016/j.juro.2011.12.105 22425048

[2] 2. Burbige KA, Hensle TW. The complications of urinary tract reconstruction. J Urol. 1986;136(1 Pt 2):292-7. 10.1016/s0022-5347(17)44845-2 3723680

[3] 3. Lima SV, Araújo LA, Vilar FO, Kummer CL, Lima EC. Nonsecretory sigmoid cystoplasty: experimental and clinical results. J Urol. 1995;153:1651-4. 10.1016/s0022-5347(01)67494-9 7715000

[4] 4. Lima SV, Araújo LA, Vilar FO. Nonsecretory intestinocystoplasty: a 10-year experience. J Urol. 2004;171(6 Pt 2):2636-39. 10.1097/01.ju.0000112782.00417.5e 15118439

[5] 5. Lima SV, Araujo LA, Vilar Fde O, Lima RS, Lima RF. Nonsecretory intestinocystoplasty: a 15-year prospective study of 183 patients. J Urol. 2008;179:1113-6. 10.1016/j.juro.2007.10.094 18206934

[6] 6. Lima SVC, Araújo LAP, Vilar FO. Oliveira, CRO., Cavalcante,NTP: Demucosalized Enterocystoplasty: Lessons learned after 20 years. J Urol. 2013;189 (Suppl):197-e198.

[7] 7. Houle AM, Gilmour RF, Churchill BM, Gaumond M, Bissonnette B. What volume can a child normally store in the bladder at a safe pressure? J Urol. 1993;149:561-4. 10.1016/s0022-5347(17)36148-7 8437265

[8] 8. Odeh RI, Farhat WA, Penna FJ, Koyle MA, Lee LC, Butt H, et al. Outcomes of seromuscular bladder augmentation versus standard ileocystoplasty: A single institution experience over 14 years. J Pediatr Urol. 2017;13:200. 10.1016/j.jpurol.2016.05.046 27576595

[9] 9. Jung HJ, Lee H, Im YJ, Lee YS, Hong CH, Han SW. Prerequisite for successful surgical outcome in urothelium lined seromuscular colocystoplasty. J Urol. 2012;187:1416-21. 10.1016/j.juro.2011.12.009 22341808

[10] 10. Jednak R, Schimke CM, Barroso U JR, Barthold JS, González R. Further experience with seromuscular colocystoplasty lined with urothelium. J Urol. 2000;164:2045-9. 11061922

[11] 11. Shekarriz B, Upadhyay J, Demirbilek S, Barthold JS, González R. Surgical complications of bladder augmentation: comparison between various enterocystoplasties in 133 patients. Urology. 2000;55:123-8. 10.1016/s0090-4295(99)00443-4 10654908

[12] 12. Hensle TW, Bingham J, Lam J, Shabsigh A. Preventing reservoir calculi after augmentation cystoplasty and continent urinary diversion: the influence of an irrigation protocol. BJU Int. 2004;93:585-7. 10.1111/j.1464-410x.2003.04664.x 15008735

[13] 13. Blaivas JG, Weiss JP, Desai P, Flisser AJ, Stember DS, Stahl PJ. Long-term followup of augmentation enterocystoplasty and continent diversion in patients with benign disease. J Urol. 2005;173:1631-4. 10.1097/01.ju.0000154891.40110.08 15821519

[14] 14. Lima SVC, Vilar FO, Lustosa ES, Aragão DCC, Calisto FCFS, Pinto FCM. New device for intermittent emptying of the bladder in female children and adolescents: A pilot study. J Pediatr Urol. 2017;13:453. 10.1016/j.jpurol.2016.12.030 28254445

